# Influence of the Fiber Post Length on the Fracture Strength of Endodontically Treated Teeth

**DOI:** 10.3390/medicina59101797

**Published:** 2023-10-09

**Authors:** Adrian-George Marinescu, Osama Abuabboud, Ștefana-Denisa Zimbru, Laura-Elena Cîrligeriu, Bianca-Adina Piț, Ioana-Amalia Borcean, Mihai Paven, Luminița-Maria Nica, Dan Ioan Stoia

**Affiliations:** 1 Department of Restorative Dentistry and Endodontics, Research Center TADERP, Faculty of Dentistry, “Victor Babeș” U.M.Ph.–Timisoara, 300041 Timisoara, Romania; marinescu.adrian@umft.ro (A.-G.M.); cirligeriu.laura@umft.ro (L.-E.C.); mihai.paven@umft.ro (M.P.); 2 Faculty of Dentistry, “Victor Babeș” U.M.Ph.–Timisoara, 300041 Timisoara, Romania; abuabboud.osama@yahoo.com (O.A.); zimbrustefana@yahoo.com (Ș.-D.Z.); pit.bianca13@gmail.com (B.-A.P.); borceanioana@gmail.com (I.-A.B.); 3 Department of Mechanics and Strength of Materials, Polytechnica University Timisoara, 300006 Timișoara, Romania; dan.stoia@upt.ro

**Keywords:** endodontically treated teeth, fiber post, post length, fracture strength, self-adhesive resin cements

## Abstract

*Background and Objectives:* Although fiber posts are widely used in the restoration of endodontically treated teeth (ETT), their ideal cementation depth into the root canal is still debated in literature. The aim of the present study was to evaluate whether the different intra-radicular insertion lengths of the fiber posts influence the fracture strength of ETT. *Materials and Methods:* A total of 10 permanent human lower incisors with straight roots of similar length and volume extracted for periodontal reason were sectioned 2 mm above the cement–enamel junction (CEJ) to a total length of 18 mm and endodontically treated in the same manner, then randomly divided into two groups of five each (Groups 1 and 2, *n* = 5). Two sound incisors, with no endodontic treatment, were used as the control group (Group 3, *n* = 2). After one week of storage in a humid environment, spaces for fiber post no. 1 (Reforpost, Angelus, Londrina, PR, Brazil) were prepared in the first two groups at a depth of 5 mm (Group 1) and 7 mm (Group 2), and the fiber posts were adhesively cemented using self-adhesive resin cement (Maxcem Elite, Kerr GmbH, Herzogenrath, Germany). After 7 days, the samples were vertically positioned and fixed in a self-curing transparent acrylic resin, up to 2 mm below the CEJ level, and mechanically tested in compression after another week of storage using a displacement-controlled testing machine up to each sample’s fracture. The force–displacement curves were recorded for each sample, the means were calculated for each group and a statistical comparative analysis between groups was conducted. *Results:* Although no statistically significant differences between groups were observed, the highest mean fracture force (N) was recorded in Group 2 (1099.41 ± 481.89) in comparison to Group 1 (985.09 ± 330.28), even when compared to the sound, non-treated teeth (1045.69 ± 146.19). *Conclusions:* Within the limitations of this in vitro study, teeth where fiber posts were placed deeper into the root canal (7 mm) recorded slightly higher fracture forces in comparison with shorter lengths (5 mm). However, similar biomechanical performances obtained in the mechanical tests showed no statistical differences between the 7 mm and the 5 mm inserted posts.

## 1. Introduction

The use of intra-radicular posts adhesively cemented into endodontically treated teeth (ETT) is needed when the amount of the remaining tooth structure is not sufficient to retain the future prosthetic restoration, as it ensures an even distribution of the masticatory forces along the root, thus preventing the tooth’s fracture [1,2]. Over time, numerous techniques and materials have been proposed for the restoration of endodontically treated teeth [3]. Compared to conventional prefabricated or cast metal materials, the advantages of root canal devices made of different types of fibers (glass, quartz, carbon), such as biocompatibility, elasticity, esthetics, or facilitating desobturation in case of retreatment, have led to their widespread use on a large scale in dentistry [4]. In addition, the combination of aesthetic and mechanical benefits of fiber devices has contributed to the development of conservative dentistry.

Whenever possible, healthy dentin should be preserved for the margins of the prosthetic restoration to rest beyond the junction of the abutment with the remaining tooth structure, thus improving the retention and strength of the tooth through the ferrule effect [5].

One of the biggest problems in restoring teeth with endodontic treatment is the risk of an irreparable fracture, which is most often attributed to the difference in rigidity between the radicular dentin and the intra-radicular devices used for restoration, thus leading to areas of stress concentration [6]. The mean value of the elastic modulus of dentin is reported to be 13.3 + 4.0 GPa, with a range of 10–30 GPa [7]. A metal post has an elastic modulus 10 times greater than that of dentin, and this mismatch in elastic modulus is believed to cause the post to debond from the root dentin [8], eventually resulting in root fracture [6]. However, glass fiber posts adhesively cemented into root canals, with a modulus of elasticity similar to the dentin, provide greater flexibility to endodontically treated teeth when subjected to occlusal forces and improve the homogeneous distribution of stress at the post–cement–dentin interface and along the radicular surface [9,10,11,12,13,14,15]. The adhesive procedure of luting the fiber posts into root canals using resin-based cements with similar mechanical properties makes the entire complex behave as a homogeneous entity during functions, preventing fracture [16,17].

Different depths of post length cemented into the root canal were studied regarding their effect on the fracture resistance of endodontically treated teeth, using either extracted teeth [1,2,6] or 3D models of human teeth [18]. In a study performed by Santos-Filho et al. [18], seven 3D models of endodontically treated incisors with intra-radicular posts of different lengths and the ferrule effect were compared to a 3D model of an intact incisor. The results showed a more homogeneous stress distribution, similar to an intact tooth, in teeth restored with fiber posts [18]. The same authors concluded that fiber glass restorations are less likely to fail, because fractures usually occur at the abutment or post level rather than on the root’s surface, and that pairing a ceramic crown with fiberglass posts showed a protective effect on the tooth’s structures. This was explained by the low modulus of elasticity and the good adhesion obtained between the ceramic, the composite abutment, and the resin cement, which creates a complex flexible restoration with mechanical properties similar to an intact tooth [18].

The idea that endodontically treated teeth reinforced with fiberglass posts have a greater longevity than those without is unanimously accepted [19,20]. The question remains whether the association between the ferrule effect and the post improves the fracture resistance of non-vital teeth, and which is the ideal length of cementation to obtain the best clinical results.

That is why the aim of the present study was to evaluate whether the different intra-radicular insertion lengths of fiberglass posts into root canals influence the fracture strength of endodontically treated teeth. The null hypothesis (H0) assumed was that there are no significant differences between different insertion lengths (5 mm and 7 mm) in comparison to sound teeth, in terms of maximum force at fracture and absorbed energy up to fracture.

## 2. Materials and Methods

This study used extracted permanent human teeth (lower incisors) from periodontal causes kept in a humid environment (saline) at room temperature from the time of extraction until the time of testing. Informed consent of the patients was obtained for extraction and for using the extracted teeth as samples in the present study. The parameters used for teeth selection in this study were single-rooted sound incisors with one straight root and only one root canal (Weine type I), of same size and volume, and the exclusion criteria concerned the existence of deep carious lesions, coronal restorations, visible fractures or cracks, previous endodontic treatments, the impossibility of performing a correct endodontic treatment, or more than one root canal into the same root. Thus, 20 teeth were initially selected, but after their visual inspection and radiological evaluation, only 12 valid teeth were selected for the present study.

All selected teeth were immersed in a 5% sodium hypochlorite solution for 15 min immediately after extraction, cleaned with an ultrasonic scaler and brushed with prophylactic paste before use. All teeth were approximately the same size and length to avoid possible errors due to volume differences. The teeth were numbered consecutively from 1 to 12. Using the simple randomized technique, the teeth were divided into 3 groups: 2 groups of 5 teeth (*n* = 5) in which the endodontic treatment was performed, and one control group of 2 sound teeth (*n* = 2). The 10 teeth belonging to groups 1 and 2 were sectioned 2 mm above the CEJ, thus all having a total measured length of 18 mm. The endodontic treatment was initiated using ISO#08-20 K-files (Kendo, VDW GmbH, Munich, Germany) for negotiation, and the root canal shaping was performed with the Wave One Gold Primary (25/.07) instrument (Dentsply Sirona, Ballaigues, Switzerland) in a reciprocating motion. The root canals were irrigated with 5.25% sodium hypochlorite (Chloraxid, Cerkamed, Starowa, Poland) for a total time of 30 min. All canals were irrigated for 3 min using 17% EDTA solution (Cerkamed, Starowa Góra, Poland), followed by saline and 96% ethyl alcohol for 1 min [21]. The canals were then dried with paper points (WaveOne Gold Primary, Dentsply Sirona, Ballaigues, Switzerland) and sealed with gutta-percha cones (WaveOne Gold Primary, Dentsply Sirona, Ballaigues, Switzerland) perfectly fitting to the taper and apical size of the preparation, in association with Adseal as endodontic sealer (Meta Biomed, Cheongju-si, Republic of Korea), using the single cone technique. All cones were sectioned below the cement–enamel junction using an electronic heated plugger (Obtura MaxPack, Obtura Spartan Endodontics, Algonquin, IL, USA) and compacted vertically with hand-held pluggers (Obtura S-Kondensors, Obtura Spartan, Fenton, MO, USA) to obtain a perfect closure of the root canal. The access cavities were cleaned with sterile cotton pellets soaked in 96% ethyl alcohol to remove any traces of gutta-percha and sealer, and the teeth were temporary sealed with a provisional material (Coltosol, Coltene Whaledent, Mahwah, NJ, USA).

After one week of storage in humid environment at room temperature, the provisional restorations were removed and the root canals of Groups 1 and 2 were prepared for glass fiber posts no. 1 (Reforpost, Angelus, Londrina, Brazil) at a depth of 5 mm in the first group (5 samples) and 7 mm in the second (5 samples), using a no. 1 calibrated drill (Reforpost, Angelus, Londrina, Brazil), corresponding to the diameter of the chosen posts. Fiber posts were then cemented according to the self-etch/self-adhesive technique: the root dentinal walls were cleaned to remove the smear layer using EDTA solution (Cerkamed, Starowa, Poland) for 1 min, washed with water, and dried with paper points and air without desiccation [21,22].

**Figure 1:**
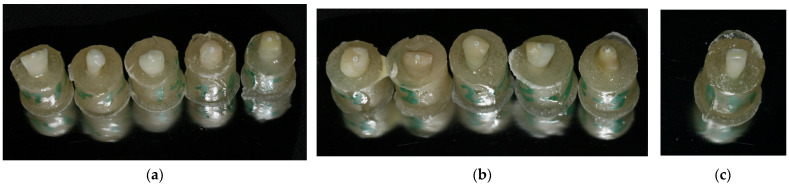
Samples from Group 1 (**a**), Group 2 (**b**) and Group 3 (**c**) ready for the mechanical testing.

The luting cement (Maxcem Elite, Kerr GmbH, Herzogenrath, Germany) was applied to the surface of the posts and injected into the root canals into the prepared post space. The posts were fixed into a vertical position in the long axis of the root canal and a light-curing unit (Valo LED, Ultradent Products Inc., South Jordan, UT, USA) with intensity of 1000 mW/cm^2^ was used for 40 s on each sample. Teeth were kept in a humid environment for another week at room temperature; afterwards, all samples, including the sound teeth of Group 3, were vertically fixed in a transparent acrylic resin cylinder (of 18 mm height and 16 mm diameter) up to 2 mm below the CEJ level (Figure 1a–c), and stored for another 7 days.

**Figure 2:**
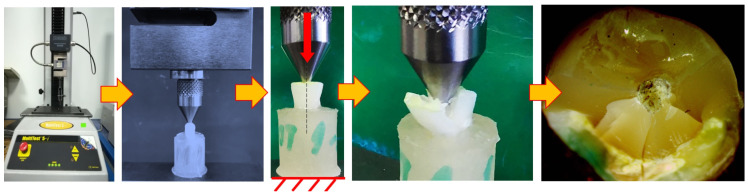
Mechanical testing of the dental samples: testing setup and result.

Each sample was afterwards removed from its environment and immediately tested for mechanical strength using a single-column tensile-compression testing machine Multitest 5i (Mecmesin, Slinfold, West Sussex, UK) equipped with a 5 kN loading cell with a measurement accuracy of ±0.1% of full scale and displacement controlled by mean of dedicated software (Emperor Force v1.18-408, Mecmesin, PPT Group, Slinfold, West Sussex, UK). Each sample was placed in the machine in a vertical position using the flat surface of the acrylic resin cylinder, on the bottom end. The loading was applied perpendicular to the center of the incisal surface of each tooth, along the axis of the root canal at the level of the fiberglass post, using a tapper steel road that ends with a rounded tip (2.5 mm radius), at a speed of 5 mm/min until the first fracture peak occurred (Figure 2).

**Figure 3:**
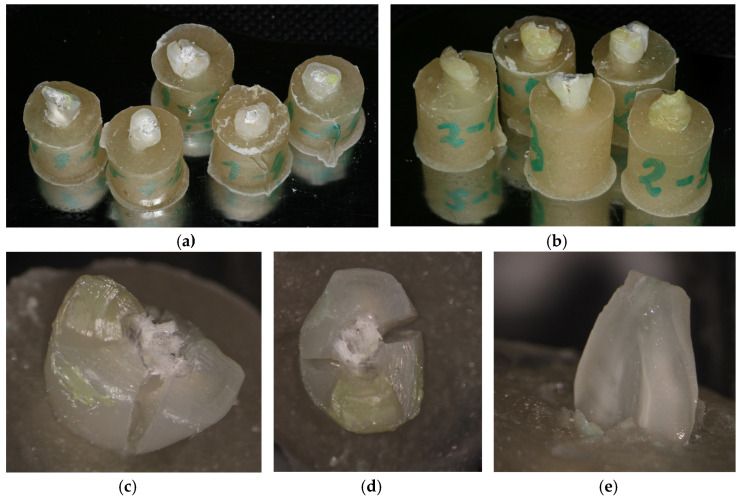
The fractured samples from Groups 1 (**a**) and 2 (**b**) after testing, and Samples 1.3 (**c**), 2.4 (**d**) and sample 3.1 (**e**) evaluated under the dental operating microscope (DOM) (×1.6).

Following their mechanical testing, the samples were visually examined to detect the destruction degree of each tooth. All fractures that occurred in the dental samples were visually analyzed under a dental operating microscope (DOM) (Alltion, Wuzhou, China) at a magnification of 1.6× and the images were recorded (Figure 3).

The absorbed energy (W) was computed as the area under the force–displacement curve for each specimen, resulting a new parameter, in Joule unit (J), that provided additional information regarding the failure of the construction [23].

In order to determine the statistical significance of the results, a one-way ANOVA single factor test with a *p*-value set as 0.05 was performed using Microsoft Excel 2021. The assumed null hypothesis (H0) was that there are no significant differences between the 3 groups. The ANOVA test was run on both fracture force and absorbed energy values of all groups.

## 3. Results

**Figure 4:**
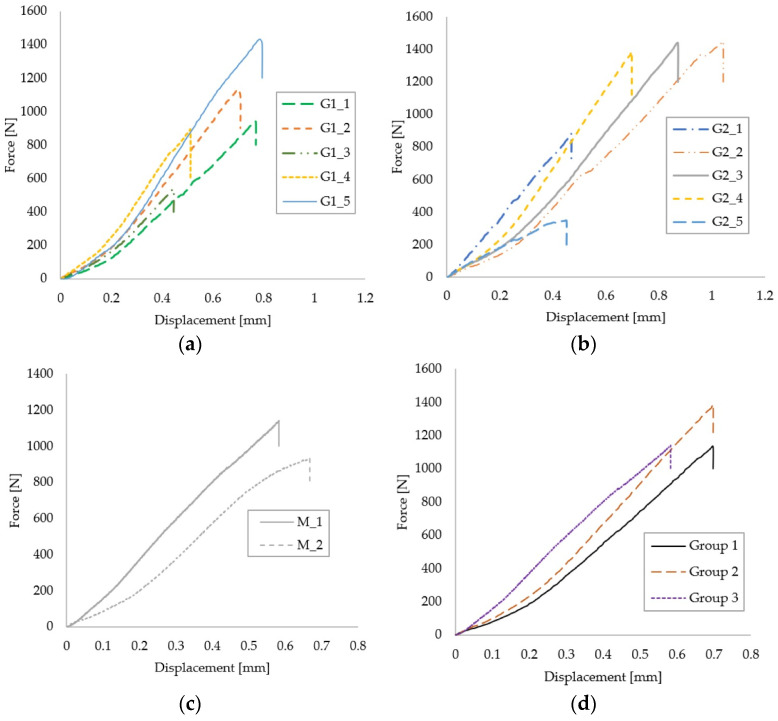
Force–displacement curves of Group 1 (**a**), Group 2 (**b**), Control Group 3 (**c**) and representative curves for each group (**d**).

The force–displacement curves of each tested sample are represented in units of N and mm, respectively, in Figure 4a–c for each group, and comparatively in Figure 4d. As can be observed, the data are relatively well grouped, taking into account the natural variability that occurs when testing biological materials or related assemblies.

**Figure 5:**
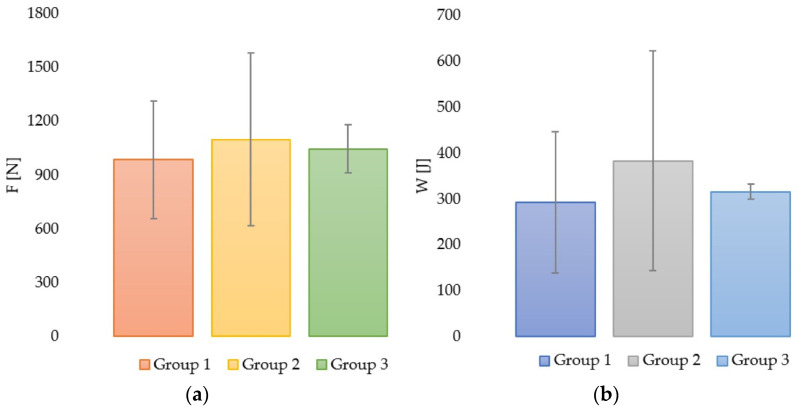
Graphical representation of fracture force (maximum force) in all groups (**a**) and absorbed energy in all groups (**b**), both with their corresponding standard deviations.

The recorded values were analyzed and compared, and the average breaking force, the standard deviation and the absorbed energy up to fracture of each group were calculated (Table 1). A graphical representation of the fracture forces and absorbed energies of Groups 1, 2 and 3 can be observed in Figure 5a,b. The average values presented here are depicted along with their standard deviations. Large variations between results can be observed for the Groups 1 and 2, compared with Control Group 3.

The lowest mean value of the fracture force was recorded in Group 1 (985.07 ± 330.28 N) in comparison to Group 2 (1099.49 ± 481.89 N) and Group 3 (1045.69 ± 146.19 N), but the standard deviation values were higher in Groups 1 and 2 in comparison to Group 3. This fact can be explained by the variable nature of the samples in the first two groups and their higher number.

The highest value of the fracture force (1447.23 N) and the highest average value (1099.41 N) were recorded in Group 2 (Table 1), but some samples from Group 1 recorded similar values of fracture force in comparison to the control group.

Also, the highest mean values for the absorbed energy were recorded in Group 2, with an average of 385.31 J, in comparison to natural, sound teeth with a mean value of 316.05 J, and Group 1 with the lowest recorded mean value of 292.03 J (Table 1).

Higher breaking energy is associated with a better performance of the construction and indicates some degree of toughness of the structure, mainly due to the fiber post.

A statistical analysis was performed to observe the significance of the differences between groups. The normality of the recorded data was first checked using the Shapiro–Wilk test with a significance level of 5%. Both groups passed the normality test, so a parametric analysis of data (ANOVA in our case) could be conducted for both fracture force and absorbed energy. The confidence interval used for testing the hypothesis was 95%. A similar approach in evaluating small samples has been used by many authors in recent publications when testing and analyzing mechanical performance (tensile tests, bending, impact, hardness, coefficient of friction) of materials for dental restoration [24], resin-modified glass ionomer [25], metal–ceramic compatibility in dental restorations [26], materials containing nanohybrid filler [27] and hypoallergenic denture base resins [28].

The results of the ANOVA single factor analysis can be observed in the Table 2. The calculated *p*-value was greater than 0.05 and F was less than F crit in both cases, which means that the null hypothesis is true and there are no significant statistical differences between the three groups in terms of fracture force and absorbed energy.

## 4. Discussion

The primary purpose of this study was to evaluate the impact that different lengths of fiberglass posts have on the fracture resistance of endodontically treated teeth. The obtained results on the mechanical testing for compression showed no statistical differences between different insertion depths of fiber posts into root canals in comparison to sound teeth. However, teeth where fiber posts were placed deeper (7 mm) recorded higher fracture values in comparison with shorter lengths (5 mm), as well as in comparison with natural, non-treated teeth. Although the lowest mean fracture force was obtained in Group 1, where fiber posts were inserted 5 mm into the root canal, all recorded forces significantly exceeded the maximum value forces that occur physiologically in anterior teeth (200 N) [29].

The results of the present study are similar to other studies in the literature. In 2009, Buttel et al. concluded that 6 mm intra-radicular posts resisted higher fracture forces than those inserted with 3 mm lengths [30]. In another study, Adanir et al. [31] reported that when using a shorter length intra-radicular post, the stress accumulation in the cervical area of the vestibular surface increases. It is unanimously accepted that a greater post length can determine a large intra-radicular accumulation of tension, but as far as fiber glass posts are concerned, because their modulus of elasticity is similar to the dentin, root fractures are less likely to occur [30,31]. Although higher fracture forces were recorded in the present study for a longer post, no statistically significant differences between groups were observed.

Even when aiming to use the maximum length of the fiberglass post, the root preparation should be as conservative as possible, thus maintaining a balance between strength and retention. In choosing the optimal length and size of the fiber post, the fracture resistance of the tooth is a more important criterion than the post’s retention in the canal. De-cementation can be resolved by applying a larger amount of cement and restoring the abutment for protection, but in the case of root fractures, the teeth are most often extracted. Tang et al. [32] showed that if an excessive amount of tooth structure is removed and the natural geometry of the root canal is altered, the stability and longevity of the tooth will be affected.

The results of this study showed that a shorter insertion length of fiberglass post led to lower values of the necessary fracture force, without being statistically different; however, the remaining dentin of the root canal walls must be conserved as much as possible. It is well known that there are other several factors that can influence the fracture resistance of a treated tooth, including the amount of remaining tooth structure, the exposure of dentin to bacteria and dehydration of the dentinal tubules [33,34]. Thus, once extracted, teeth dehydrate quite quickly, which may negatively influence the results of mechanical testing. That is why teeth were kept in a humid environment in all the steps of the present experimental study.

Normally, after an endodontic treatment and the insertion of a post, a prosthetic treatment with crown follows. In the present study, teeth were not protected with crowns before mechanical testing, so the real clinical situation of a patient could not be imitated. Lin et al. [35] developed an experimental study on 48 endodontically treated maxillary teeth. All teeth were prepared with glass fiber posts, with different combinations of post lengths (7.5, 11 and 15 mm) and abutment heights (3 and 5 mm), and afterwards restored with full zirconia crowns. The results showed that the highest mean fracture resistance was recorded for the 15 mm post length and 5 mm abutment height test group, which was significantly more resistant to fracture than the 7.5 mm post and 5 mm abutment height group. Thus, the study concluded that increasing the post length inside the root canal of the endodontically treated teeth restored with glass fiber posts leads to an increase in fracture resistance, which is similar to our test results [35]. The selected depths for the fiber post insertion in the present study were 7 mm, similar to one of the groups tested by Lin et al. [35], and 5 mm, which corresponds to a length equal to an abutment height of 5 mm.

In another study performed by Schiavetti and Gianino [36] on human extracted premolars, fiber post depths of 5, 7 and 9 mm were mechanically tested for fracture resistance; their conclusion was that the insertion depth of the post did not statistically influence the resistance of the treated teeth. These results are similar to our current findings, using only two groups with similar post length (5 and 7 mm). Also, the ferrule effect of the tested teeth in the above cited study was 2 mm, which is exactly the one selected when preparing the samples to be tested in the present study.

There are situations where the use of a smaller post is indicated: when the ideal length of the post can compromise the apical seal, a shorter post must be used. In this case, the shorter post length will be compensated by the higher retentive capacity of the luting cement, the cementation of the fiber post being an adhesive procedure and not just one based on mechanical retention. Even if a longer fiberglass post is preferred, at least 3 to 6 mm of apical seal must be maintained, and the root canals must not be excessively and unnecessarily prepared [34].

The post cemented into the root canal performs both a mechanical and biological function by protecting the apical seal from bacterial infiltration in case of coronal leakage [37], functions which should not compromise the tooth strength. That is why the post space preparation should conserve as much as the remaining radicular substance, avoiding an increased risk of root fracture [37]. As the retention of fiber posts is based on adhesion, one-third to one-half maximum of the length of the root canal is recommended to be desobturated for post space preparation and cementation depth [37,38,39]. Also, the radicular extension of the post should be about the coronal length of the core [37,38,39]. As observed in the present study, the selected cementation lengths of 5 and 7 mm for the fiber posts fulfil these criteria and recommendations, as these lengths represent exactly a third or half of the length of the roots in the selected samples. Also, the 5 mm radicular extension of the posts in Group 1 is equal to the coronal length of an ideal abutment [35,37,38,39].

The use of self-adhesive resin cements (SARCs) in the luting procedure of the fiber posts, as the cement selected for the present study, offers the advantage of a single step clinical procedure to achieve the cementation of the fiber posts into the root canal, proving similar bonding results to multistep luting cements [40]. Although dual-cured, the recommendations for these cements are to be light activated at the end of the luting procedure to achieve superior material properties [41]. That is why all samples tested in the present study were light-cured for 40 s, as described in the Section 2.

The height of the remaining sound tooth structure is also important. Several studies recommend an ideal height of 2 mm intact tooth structure above the restoration margins to ensure a proper ferrule effect [42,43,44]. For this reason, all dental samples included in this study were sectioned 2 mm above the CEJ, so the ferrule height was 2 mm in all tested treated samples, which may explain the similar values between the control group and the two other groups. The increased amount of remaining structure allows a better distribution of forces. Moreover, the more coronal structure remaining, the better the retention between the post and the tooth [45]. The ferrule effect of 2 mm in all samples of the present study and the restoration of teeth with fiber posts led to a similar fracture resistance of the ETT in comparison to sound teeth, as no statistically significant differences were recorded.

However, the limitations of in vitro test should be mentioned, as they only partially reflect clinical situations. Intraoral conditions could not be exactly reproduced in the present study in terms of thermal and chemical changes, or the cyclicity of masticatory forces, and tested teeth were not restored with prosthetic crowns.

Because of the many variables involved, including the limited number of tested teeth and the differences in the mechanical properties of the dentin, it is almost impossible to compare the clinical fracture resistance with the present in vitro study. In fact, the most unpredictable factor is the condition of the tooth, which is dependent on the quality and quantity of its remaining dentin. This is also the main disadvantage of using extracted human teeth, as it may lead to a larger standard deviation, so our results should be interpreted in light of these limitations.

## 5. Conclusions

Based on the results of the present study and within its limitations, it can be concluded that teeth where fiber posts were placed deeper into the root canal (7 mm) recorded slightly higher fracture forces in comparison with shorter lengths (5 mm). However, similar biomechanical performances obtained in the mechanical tests showed no statistical differences between the 7 mm and the 5 mm inserted posts. The correct insertion of the fiber post must be associated with the preservation of the remaining hard tooth structure. Posts should be placed without weakening the apical seal, excessively removing the root canal dentin, or producing perforations.

## Figures and Tables

**Table 1:** Maximum force (F_max_, N), displacement at maximum force (δ at Fmax, mm) and absorbed energy (W, J) for each group.

SAMPLE	GROUP 1			GROUP 2			GROUP 3		
	F_max_	δ at F_max_	W	F_max_	δ at F_max_	W	F_max_	δ at F_max_	W
1	1430.81	0.69	349.74	1447.23	1.04	677.29	1141.25	0.58	327.96
2	1132.25	0.44	98.21	1437.52	0.87	535.41	950.14	0.66	304.13
3	938.19	0.51	201.92	1383.78	0.69	413.95	-	-	-
4	891.92	0.78	505.80	880.72	0.45	88.58			
5	532.16	0.76	306.27	347.81	0.47	202.32			
AV	**985.09**		**292.39**	**1099.41**		**383.51**	**1045.69**		**316.05**
SD	**330.28**		**154.04**	**481.89**		**239.85**	**146.19**		**16.84**

**Table 2:** The ANOVA single factor analysis for fracture force and absorbed energy.

**SUMMARY**		*Fracture force data*			*Absorbed energy data*		
*Groups*	*Count*	*Sum*	*Average*	*Variance*	*Sum*	*Average*	*Variance*
1	5	4925.36	985.09	109,092.5	1461.96	292.39	23,729.22
2	5	5497.08	1099.41	232,223.4	1917.57	383.51	57,530.04
3	2	2091.39	1045.69	18,261.5	632.10	316.05	283.91
**ANOVA**							
*Source of Variation*		*SS*	*df*	*MS*	*F*	*p-value*	*F crit*
*Fracture force data*							
Between Groups		32,695.35	2	16,347.67	0.106344	0.900229	4.256495
Within Groups		1,383,525	9	153,725			
Total		1,416,221	11				
*Absorbed energy data*							
Between Groups		21,558.15	2	10,779.07	0.298203	0.749208	4.256495
Within Groups		325,321	9	36,146.78			
Total		346,879.1	11				

## Data Availability

Data available on request from the corresponding author.
